# The Effect of Grape Seed Extract on the Alveolar, Jaw, and Skeletal Bone Remodeling: A Scoping Review

**DOI:** 10.1055/s-0043-1768975

**Published:** 2023-06-13

**Authors:** Erdiarti Dyah Wahyuningtyas, Ari Triwardhani, I Gusti Aju Wahju Ardani, Meircurius Dwi Condro Surboyo

**Affiliations:** 1 Department of Orthodontic, Faculty of Dental Medicine, Universitas Airlangga, Surabaya, Indonesia; 2 Department of Oral Medicine, Faculty of Dental Medicine, Universitas Airlangga, Surabaya, Indonesia

**Keywords:** grape seed extract, proanthocyanidins, bone remodeling, human health

## Abstract

Herbal medicine has an important part in promoting and maintaining human health. One of them was grape seed extract (GSE). Various potentials of GSE in human health have been explored, and its potential for maintaining bone health is promising. Some initial research has provided evidence that the GSE was able to affect bone remodeling (bone resorption and bone formation). This scoping review analyzed and discussed all the reports on the effect of GSE on bone healing and bone remodeling in animals in the alveolar bone, jaw bone, and skeletal bone. The further purpose is to give an opportunity to research and development of supplementation of GSE for humans.

The Preferred Reporting Items for Systematic Reviews and Meta-Analysis (PRISMA) 2020 guidelines were used to compose this scoping review through database on Scopus, PubMed, Science Direct, Web of Science, Embase, and manual search until December 2022. The inclusion criteria were a study that analyzed the effect of supplementation GSE on all bones.

All included study was *in vivo* study with supplementation of GSE. The supplementation of GSE affects the alveolar bone, jaw bones, and skeletal bone by promoting bone formation and inhibiting bone resorption by suppressing inflammation, apoptosis pathways, and osteoclastogenesis. It not only supports bone remodeling in bone inflammation, osteonecrosis, osteoporosis, and arthritis but also the GSE increases bone health by increasing the density and mineral deposition in trabecula and cortical bone.

The supplementation of GSE supports bone remodeling by interfering with the inflammation process and bone formation not only by preventing bone resorption but also by maintaining bone density.

## Introduction

In recent years, the grape has explored its bioactive compounds, such as proanthocyanidins and phenolic acid, that are found in the skin, pulp, and seed. 1 The grape seed extract (GSE) has attracted the attention of the food industry and public health organizations. 2 GSE has potential health benefits because of rich proanthocyanidins, 3 approximately 74 to 78%, 4 and it possesses anti-inflammatory, antioxidant, 5 antiapoptotic and pro-proliferative properties. 6 Besides its potential, the GSE is considered safe for humans, because it does not show any toxicity effects like changing the hematological parameter and organ changes. 7 By that A GSE has been recognized by the Food and Drug Administration as Generally Recognized as Safe and is sold commercially as a dietary supplement, and is listed in the Everything Added to Food in the United States data. 8

In dentistry, the GSE has been proved to prevent dental caries 9 10 by covering the acquired enamel pellicle and preventing bacterial adhesion to performed biofilm 11 and antibacterial in the root canal during endodontic treatment. 12 The GSE also has been promoted as periodontitis medication because it is able to reduce oxidative stress and inflammation. 13 In clinical randomized clinical trials, the GSE significantly reduced the probing depth and increased the attachment level. 14 Recent research has shown GSE's influence in bone remodeling process in postorthodontic relapse prevention by inhibiting osteoclastogenesis. 15 Bone remodeling is an active and dynamic process between bone resorption by osteoclasts and bone formation by osteoblasts that works in balance to maintain mineral homeostasis in the body. 16 It is no exception that prevention of postorthodontic relapse also requires adequate bone remodeling, which is an important factor in maintaining bone thickness. During orthodontic tooth movement, bone resorption occurs in the pressure area, due to osteoclast activation, and bone formation in the pull area, due to osteoblast formation. 17 Not only alveolar bone remodeling but also the changes that include periodontal ligament metabolism 18 and neural regulation occurr. 19 This process should occur in a balanced manner until the teeth on the arch is achieved. The undesirable thing is that excessive resorption occurs without being followed by the bone formation in the tension area of the teeth involved. More importantly, the height of the alveolar bone and the thickness of the cortical bone must be maintained. 20

The current data showed that with the consumption of 200 to 400 mg per day of GSE as food supplementation, no physiological or clinical abnormality was changed, and it was declared safe for consumption. 21 The safety is related to high proanthocyanidins and safe to gastrointestinal mucosa. 22 23 The proanthocyanidins exerted an antioxidant and anti-inflammatory effect by inhibiting the production of a pro-inflammation cytokine through the inhibition of nuclear factor kappa B (NF-kB) 24 and the C-reactive protein (CRP). 25 26 Recent research also provided that the supplementation of GSE containing high proanthocyanidins has great benefits for humans by providing antioxidative stress and anti-inflammation, 27 improving bone health such as preventing bone loss, 28 inhibiting bone resorption by inhibiting osteoclastogenesis through NF-kB and c-Jun N-terminal kinase (JNK) signaling, 29 inhibiting advance glycation end product, 30 increasing bone formation by increase bone mineral and density, 31 upregulating bone growth factors, such as bone morphogenetic protein 7 (BMP-7), 30 and increasing implant osseointegration. 28 On the other hand, the GSE also provides a protective effect against osteoarthritis in the knee. 32 With various potentials regarding GSE for various bone remodeling markers, due to limitations and explanations of the exact mechanism of GSE for alveolar bone, jaw bone, and skeletal bone remodeling, especially in treatment-related like postorthodontic relapse prevention, dental implant, periodontal treatment, or dental surgery, this scoping review was conducted to further explain the mechanism and provide an opportunity for further research to be performed.

## Methods

### Review Methodology

This scoping review of published studies on the effect of GSE on bone remodeling was conducted according to the Preferred Reporting Items for Systematic Reviews and Meta-Analysis (PRISMA) 2020 guidelines. The focus question in this review was, “How does GSE affect bone remodeling (alveolar bone, jaw bone and skeletal bone)?

The Population, Intervention, Comparison, Outcome statement used for this study is the population included in all the studies investigating the potential effect of GSE on bone remodeling, including alveolar bone, jaw bones, and skeletal bone. Intervention is defined as the various dose and administrations of GSE. Any comparison (placebo or no control) was included. All clinical outcomes related to bone remodeling markers by *in vivo* research were included.

### Information Sources

A comprehensive literature search was conducted on the following databases: PubMed, Scopus document, Science Direct, Web of Science, Embase, and manual search for all studies published.

### Search Strategy

The keyword as [(grape seed) or (grape seed extract)] AND [(bone) or (bone remodeling)] were used in the research. Results were limited to studies published in English and *in vivo* studies. Review articles were not included in this review.

### Study Design and Selection Process

All studies on those databases and fitting the criteria below were grouped together, and any duplicates were removed. The remaining studies were then filtered according to the title and the abstract. Studies that did not match were excluded at this stage. The remaining studies were screened at the final stage according to their full text, and those that did not meet the inclusion criteria were excluded. *Mendeley reference manager* was used to organize the study titles and abstracts and identify duplicates. 33 This process was conducted by four independent investigators: EDW, AT, IGAWA, and MDCS. In the case of disagreements, the investigators reached their decision through discussion.

The inclusion criteria for this review included clinical or *in vivo* studies about GSE, studies describing its potential effect on bone remodeling, the dose of the treatment, and the marker analyzed. This process, documented by Microsoft Excel for Windows, was performed in the following order: the name of the first author, publication year, country, study design, and results.

## Results

### Study Selection

**Fig. 1:**
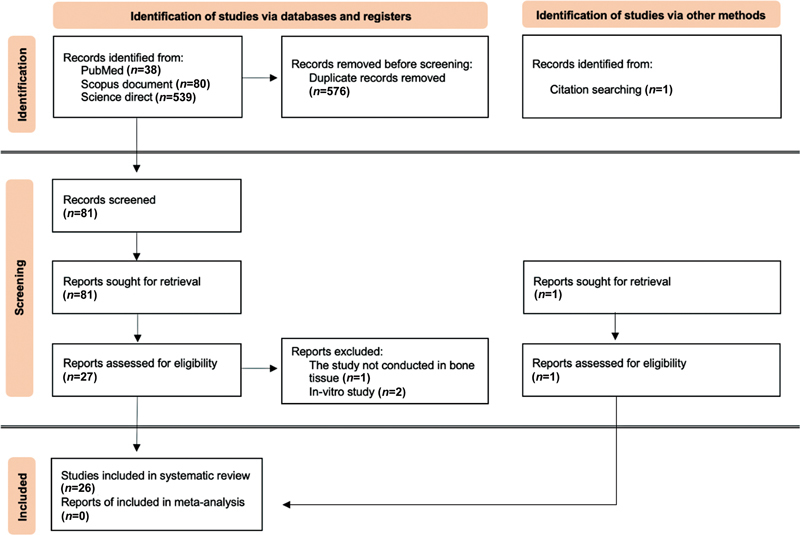
Preferred Reporting Items for Systematic Reviews and Meta-Analysis (PRISMA) flowchart.

After using a combination of keywords, 659 articles were found in the three databases. The titles were screened, and the duplicates were removed, resulting in 82 remaining articles. After reading the abstracts, all 82 articles were included in the next step of assessing the full text for eligibility. After this process, only 26 articles analyzed the effect of supplementation of GSE to the alveolar bone (7 articles), jaw bones (4 articles), skeletal bone (long and flat bone) (6 articles), and in the bone disease model (9 articles; Fig. 1 ).

### Study Characteristics

Twenty-six studies revealed the potential effect of GSE on the alveolar bone, including maxilla, 15 interpremaxillary, 34 alveolar bone in a molar area, 35 36 37 and incisive area 38 39 the mandibular jaw bone 40 41 and condyle 31 42 skeletal bone including the femur, 28 43 calvaria, 28 and tibia. 28 44 45 46 47 In the disease model, femur, 48 49 50 51 52 tibia, 53 54 and knee were used. 55 56

The study of supplementations of GSE was mostly performed in Wistar rats, 15 31 35 36 37 38 40 41 42 43 44 45 46 47 49 50 54 55 Sprague-Dawley rat, 34 rabbits, 39 51 52 and mice. 28 48 56

### The GSE Effect on Alveolar Bone

The GSE was administered per-orally in various doses, ranging from 0.1 mL, 0.5 mL.kg, 12.5 mg/mL, 50 mg/kg, 100 mg/kg, and 200 mg/kg. The orthodontic intervention was performed like coil springs, helical springs, and orthodontic wire. The periodontal intervention related to alveolar bone was placed with silk and a braided suture in the cervical of the teeth. And in other studies, tooth extraction was performed.

All the interventions showed increased bone remodeling, marked with increased osteocalcin and osteoblast; decreased receptor activator of NF-kB ligand (RANKL), osteoprotegerin (OPG), osteoblast; decreased inflammatory responses marker with decreased serum malonaldehyde (MDA) and gingival tissue level, inflammatory cell, matrix metalloproteinase 8 (MMP-8) and hypoxia-inducible factor 1α (HIF-1α); increased anti-inflammatory response marked by an increase in the glutathione (GSH) level; and decreased alveolar bone resorption and alveolar bone loss marked by decreased osteoclast and increased bone morphological protein 2 (BMP-2; Table 1 ).

**Table 1:** The effect of GSE administration on the alveolar bone of animals

Animals	Bone location	Bone intervention	GSE treatment		Comparison	Treatment outcome	References
			Doses	Duration			
Rat—Wistar	Maxillary central incisive	An orthodontic force with Stainless steel 3-spin coil spring	12.5 mg/mL	Once a day for 1/3/7/14 days	Without GSE treatment	Lower osteoclast number	15
Rat—Sprague-Dawley	Inter-premaxillary suture	An orthodontic force with helical springs steel wire	100 mg/kg	15 days	Without GSE treatment	Higher new bone formation	34
Rat—Wistar albino	Mandibular first molar	Ligature induced periodontitis using 0.5 mm orthodontic wire	50 mg/kg	Once every 3 days for 1/7/28 days	Saline solution	Lower MDA serum level	35
						Lower MDA gingival tissue level	
						Higher GSH level	
						Lower inflammatory cells	
						Lower alveolar bone resorption	
Rat—Wistar	Mandibular first molar	Ligature induced periodontitis using silk suture	100 mg/kg 200 mg/kg	Once a day for 30 days	Saline solution	Higher number of osteoblasts	36
						Lower number of osteoclasts	
						Lower alveolar bone loss	
						Lower MMP-8 expression	
						Lower HIF-1α expression	
Rat—Wistar	Maxillary first molar	Ligature induced periodontitis braided silk	50 mg/kg 100 mg/kg	Once a day for 14 days	Without GSE treatment	Lower alveolar bone loss	37
						Higher osteocalcin	
Rat—Wistar	Mandibular first incisor	Tooth extraction	0.1 mL	Once time	Without GSE treatment	Higher of osteoblast	38
Rabbit—New Zealand	Maxillary first incisor	Tooth extraction	0.5 mL/kg	Once time	Hemostatic sponge	Higher of BMP-2	39

Abbreviations: BMP-2, bone morphogenetic protein 2; GSE, grape seed extract; HIF-1 α, hypoxia-inducible factor 1α; MDA, malonaldehyde; MMP-8, matrix metalloproteinase 8.

### The GSE Effect on Jaw Bone

The supplementation of GSE affected mandibular bone by 3 mg dose once a week for 3 and 6 weeks. The cortical and trabecular bone marked an increase in density and bone mineral content, calcium, phosphate, and improved bone strength ( Table 2 ).

**Table 2:** The effect of GSE administration on the jaw bone of animal

Animals	Bone location	Bone intervention	GSE treatment		Comparison	Treatment outcome	References
			Doses	Duration			
Rat—Wistar	Mandibular	Standard diet with GSE supplementation	3 mg	21 days	Standard diet	Higher trabecular high density	40
						Higher bone mineral content	
						Higher cortical bone density	
						Higher bone mineral content	
						Higher bone strength	
Rat—Wistar	Mandibular condyle	Standard diet with GSE supplementation	3 mg	21 days	Standard diet	Higher bone cortical density	31
						Higher bone total density	
Rat—Wistar	Mandibular condyle	Low calcium diet with GSE supplementation	3 mg	21 days	Low calcium diet	Higher cortical bone density	42
						Higher trabecular bone mineral content	
Rat—Wistar	Mandibular	Combination low and high calcium diet with GSE supplementation	3 mg	42 days	Combination low and high calcium diet	Higher cortical bone density	41
						Higher trabecular bone mineral content	

Abbreviation: GSE, grape seed extract.

### The GSE Effect on Skeletal Bone

The supplementation of GSE affected the femur, calvaria, and tibia bone with osteotomy, defect and implant placement. The dose was 10 to 100 mg/kg. The bone remodeling showed bone healing and improvement after the defect, increased callus formation, bone volume, and increased torque of implant removal ( Table 3 ).

**Table 3:** The effect of GSE administration on the skeletal bone of an animal

Animals	Bone location	Bone intervention	GSE treatment		Comparison	Treatment outcome	References
			Doses	Duration			
Wistar rats—Albino	Femur shaft	Osteotomy	100 mg/kg	10/20/30 days	Nonfracture and standard diet	Higher bone improvement	43
						Higher bone healing	
						Higher bone strength	
Mice—C57BL/6 J	Calvaria	Bone defect	10 mg/mL/kg	13 weeks	Pure water	Higher bone density	28
	Femur					Lower bone defect volume	
	Tibia	Implant placement				Higher new bone formation	
						Higher removal torque of implant	
Rat—Wistar	Tibia	Combination standard diet and low calcium diet and GSE supplementation	3 mg	3 weeks	Combination standard diet and low calcium diet and tap water	Higher trabecular bone density	44
						Higher trabecular bone mineral	
Rat—Wistar	Tibia	Combination standard diet and low calcium diet and GSE supplementation	3 mg	3 weeks	Combination standard diet and low calcium diet and tap water	Higher cortical bone mineral	45
						Higher calcium and phosphate content	
Rat—Wistar	Tibia	Combination standard low and high calcium diet and GSE supplementation	3 mg	3 weeks	Combination standard low and high calcium diet and tap water	Higher trabecular bone density	46
						Higher trabecular bone mineral	
Rat—Wistar	Tibia	Combination standard low and high calcium diet and GSE supplementation	3 mg	3 weeks	Combination standard low and high calcium diet and tap water	Higher cortical cone density	47
						Higher cortical bone mineral	
						Higher bone strength	

Abbreviation: GSE, grape seed extract.

### The GSE Effect on the Skeletal Bone with Disease

The supplementation of GSE also showed a good response for bone remodeling in bone diseases like bone inflammation by lipopolysaccharide (LPS), osteonecrosis, arthritis, and osteoporosis. GSE doses vary from 12 mL/kg to 300 mg/kg.

The GSE increased the Rcan 3, Runx2, and Sox6 expressions, osteocalcin, phosphor and calcium content, bone volume and density, and also thickness of trabecula during bone formation. However, the GSE also inhibited bone resorption through a decreased number of osteoclast and inflammation process. The inflammation process reduced the 8-oxo-2'-deoxyguanosine, Superoxide dismutase (SOD), Glutahione (GSH), Malondialdehyde (MDA), Caspase 3, and interleukin-1β (IL-1β) values. While it was related to bone destruction, it decreased nitro tyrosine, RANK, NFATc1, LRP, Tcf3, and MMP-13 expression ( Table 4 ).

**Table 4:** The effect of GSE administration on the bone disease model of animal

Animals	Bone location	Bone intervention	GSE treatment		Comparison	Treatment outcome	References
			Doses	Duration			
Mice—ICR	Femur	Bone inflammation with LPS	200 mg/kg	Once time a day for 8 days	PBS	Lower number of osteoclasts	48
						Higher bone density	
						Higher trabecular thickness	
						Higher trabecular number	
Rat—Albino	Femur	Osteoporosis model with dexamethasone	400 mg	Three time per week for 4 weeks	Without GSE treatment	Improve bone structure	49
Rats—Y59 growing	Femur	Osteoporosis model with retinoic acid	100 mg/kg	Once a day for 14 days	Water or alendronate	Increase trabecula formation and thickness	50
						Increase bone mineral content and density	
Rabbits - New Zealand white	Femur	Osteonecrosis model induced by high-dose methylprednisolone	12 mL/kg	Once a day for 14 days	PBS	Lower bone necrosis	51
						Lower 8-oxo-2'-deoxyguanosine	
						Lower SOD	
						Lower GSH levels	
						Lower MDA levels	
						Lower apoptosis index	
						Lower caspase 3	
Rabbit—Japanese white	Femoral head	Osteonecrosis model with *Escherichia coli* endotoxin and methylprednisolone	200ug/kg	3 times every 24 hours	Saline solution	Increase Bcl2 expression	52
						Decreased caspase 9 expression	
Mice—DBA/1J	Tibiotalar joint of the ankle	Arthritis model with complete Freund's adjuvant	100 mg/kg	3 times at the interval 24 hours	Without GSE treatment	Higher SOX6 expression	53
						Higher RunX2 expression	
						Higher Rcan 3 expressions	
						Lower NFATc1 expressions	
						Lower nitro tyrosine expression	
						Lower RANK expressions	
						Lower LRP-4 expressions	
						Lower Tcf3 expressions	
Rat—Wistar	knee joint	Arthritis model with sodium iodoacetate	100 mg/k	Twice weekly for 18 days	Saline solution	Lower MMP-13 expressions	54
						Lower nitro tyrosine expressions	
						Lower IL-1β expressions	
						Lower number of osteoclasts	
						Increase phosphor and calcium content	
						Increase osteocalcin	
Rat—Wistar	Knee joint	Arthritis by monoids acetate	200 mg/kg 400 mg/kg	once a day for 10 days	Without GSE treatment	Reduce bone loss	55
Mice—DBA/1J	Knee joint	Arthritis model with complete Freund's adjuvant	10 mg/kg 50 mg/kg 100 mg/kg	5 times per 2 days for 2.5 weeks	Saline solution	Reduce the osteoclast	56
						Decreased the TNF-α	
						Decreased the IL-17	

Abbreviations: GSE, grape seed extract; GSH, glutathione; ICR, Institute of Cancer Research; IL-7, interleukin-7; LPS, lipopolysaccharide; LRP-4, lipoprotein receptor-related protein 4; MDA, malonaldehyde; MMP-13, matrix metalloproteinase 13; PBS, Phosphate buffer saline; TNF-α, tumor necrosis factor-alpha.

## Discussion

Various studies have been reported regarding the potential of GSE for human health. GSE supplementation with the main content of proanthocyanidins is widely used to treat obesity, 57 especially when it comes to weight control, 26 blood glucose, 58 cholesterol, 59 and blood pressure. 60 61 62 GSE supplementation can also be used to improve and prevent cardiotoxicity, gastrointestinal toxicity, hepatotoxicity, nephrotoxicity, and mucositis caused by cancer radiation, like. 63 In inflammation, GSE can significantly inhibit the formation of CRP. 64 As a natural ingredient that is safe for consumption, GSE is also proven to be safe for the liver because it can improve levels of alanine aminotransferase, aspartate aminotransferase, and alkaline phosphatase. 65 In addition, GSE is also able to provide antibacterial properties. 66

With various studies on the potential of existing, it seems that the potential for bone health has not been widely disclosed, so application and testing in humans have not been widely performed. Various *in vivo* studies have shown a lot of GSE potential for bone remodeling processes in the alveolar bone, jaw, and skeletal bones. One of the potentials of GSE for bone remodeling in the alveolar bone can be seen in various treatments in the field of dentistry, such as orthodontic, periodontal surgery, dental implant, or oral surgery.

**Fig. 2:**
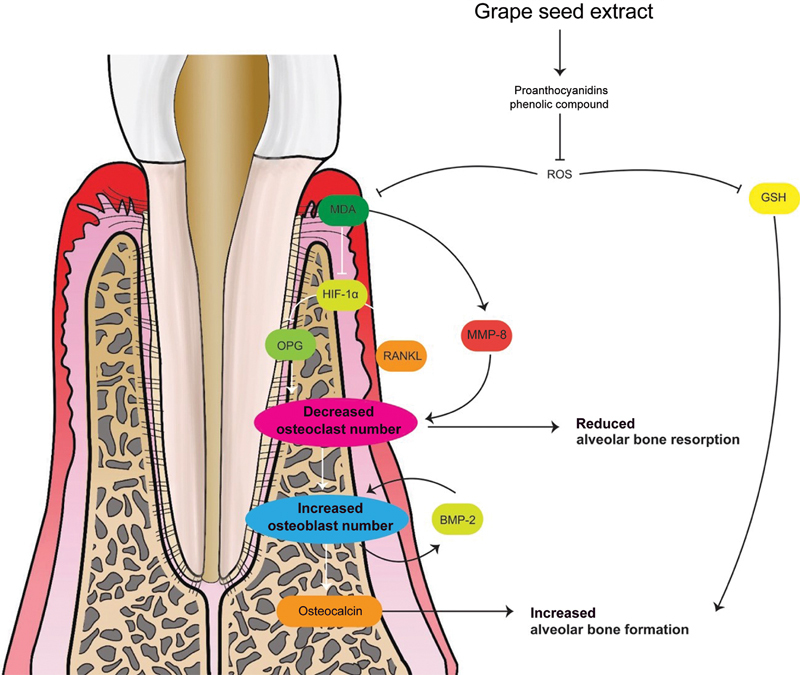
The possible mechanism of grape seed extract supplementation for alveolar bone remodeling. BMP-2, bone morphogenetic protein 2; HIF-1α, hypoxia-inducible factor 1α; MDA, malonaldehyde; MMP-8, matrix metalloproteinase 8; OPG, osteoprotegerin; RANKL, receptor activator of nuclear factor-kB ligand; ROS, reactive oxygen species.

During orthodontic treatment, tooth movement is strongly influenced by the processes of resorption and formation of the alveolar bone. GSE supplementation in the prevention of post orthodontic relapse provides an anti-inflammatory effect by decreasing the production of MDA in serum and gingival tissue. 35 This decrease occurs because phenolic compounds in GSE can inhibit the formation of reactive oxygen species (ROS) and the formation of MDA. 67 The further possible mechanism was explained through periodontitis-related alveolar bone resorption. The MDA maybe then decrease HIF-1α and MMP-8 expression, 36 resulting in a decrease in the inflammatory response, 35 and several osteoclasts. 15 36 HIF-1α is one of the important factors in osteoclastogenesis, especially the hypoxia response that occurs in orthodontics tooth movement 68 and a factor in osteoclast activation, 69 through the increased of OPG secretion to bind to RANKL. 70 On the other hand, GSE is also able to reduce RANKL and OPG, thereby reducing the number of osteoclasts 15 36 and increasing the osteoblast, 36 which results in an excessive decrease in alveolar bone resorption 35 or bone loss. 37 Further, the mechanism of GSE to support bone formation was explained in the tooth extraction model, where the alveolar bone expressed increased osteoblast, and the bone growth factor was BMP-2 38 39 and osteocalcin for alveolar bone mineralization 36 ( Fig. 2 ).

The antioxidant properties of GSE are also responsible for increasing the formation of GSH in serum, 35 thus supporting the formation of osteoblasts, 36 and maintaining the height of alveolar bones and preventing bone loss. 34 36 This increase in GSH is due to the inhibition of ROS by the phenolic compound in GSE, which then affects the NF-κB signal pathway involved in osteoclast differentiation 71 ( Fig. 2 ).

**Fig. 3:**
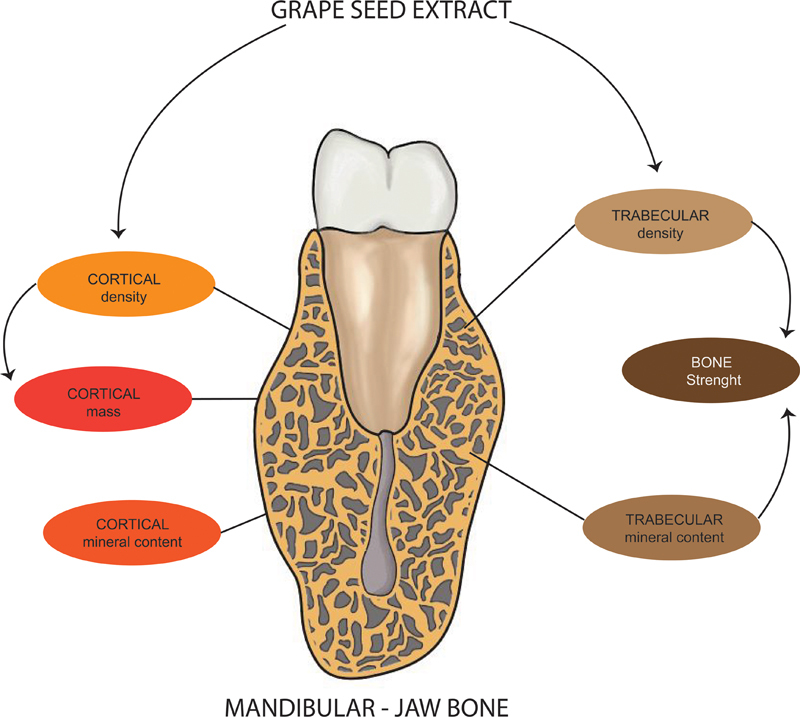
The possible mechanism of grape seed extract E supplementation for mandibular bones related to its strength.

Although the exact mechanism of how GSE can affect bone remodeling is still unknown, some previous studies have confirmed this. GSE *in vivo* level research has been shown to affect mandibular bone 40 41 and mandibular condyle. 31 42 GSE supplementation for 3 to 6 weeks has been shown to increase trabecular density 31 40 and cortical density 40 41 42 45 and is implicated in increasing bone strength ( Fig. 3 ). Analysis of bone content also showed that levels of minerals, 40 41 calcium and phosphate in mandibular bones, 41 45 were significantly increased in the group that received GSE supplementation. The explanation of increasing jaw density and minerals, calcium, and phosphate is not fully understood.

Adequate bone remodeling is also needed during bone recovery due to bone disease. GSE supplementation was performed in some studies related to bone healing-related disease. The *in vivo* model was performed by LPS to induce inflammation model, 48 osteoporosis, 49 50 osteonecrosis, 51 52 and arthritis. 53 54 55 56 In the bone inflammation and osteoporosis model, GSE maintained the bone structure, 49 by increasing the trabecular thickness 48 50 and bone mineral content. 50 The exact mechanism decreased the number of osteoclasts. 48 The proanthocyanidins are responsible for this mechanism because this active substance is able to inhibit the osteoclast through inhibition of activation of NF-kB and JNK signaling pathways. 29

In the osteonecrosis model, the antioxidant properties of proanthocyanidins in GSE take place by controlling the radicals like SOD, 51 GSH, 51 MDA, 51 and pro-apoptosis proteins like caspase 3, 51 caspase 9, 52 and Bcl2. 52 It has been researched that proanthocyanidins can inhibit mitochondrial stress and prevent the apoptosis process by inhibiting the intrinsic apoptosis pathways. 72 In the orthodontic field, the force applied will activate the hypoxia, produce ROS, and activate the NF-kB, 71 which may increase alveolar bone resorption for tooth movement. For this reason, the supplementation of GSE to prevent post-orthodontic tooth movement (relapse) needs to be explored.

In the arthritis model, the GSE plays an antioxidant property in preventing bone destruction and inflammation through increased Sox6 expression. The SOX6 expression takes place during bone remodeling in the arthritis model. 53 Sox6 is the major factor for healing because it is able to enhance proliferation, inhibit apoptosis, and regulate osteogenesis-related gene expression. 73 The sox family, Sox5, Sox6, and Sox9, is involved in the activation and maintenance of chondrogenesis during fracture healing and the enhancement of chondrogenesis by BMP-2 74 further Sox6 expression also determined bone mineral density. 75

The other protein that influences bone remodeling is Runt-related transcription factor 2 (Runx2). 76 This protein is essential for osteoblasts and osteoclasts differentiation. 77 Some research also mentions that upregulated Runx2 and Sox6 also contribute to bone formation, especially for chondrocyte differentiation. 78 Related to increased runx2 expression after supplementation of GSE on the arthritis model, 53 the supplementation also decreased the HIF-1α expression in the alveolar bone. But the relationships between Runx2 and HIF-1α have been explained by Lin et al., 2011, in which the inhibition of Runx2 and HIF-1α resulted in heterotopic ossification forming. 79 In alveolar bone, the Runx2 also plays similar as skeletal bone, which is a role in osteoblast differentiation 80 and maintains the integrity of the dentogingival junction. 81

NFATc1 expression also decreases after GSE supplementation in the arthritis model. 53 During the regeneration, GSE provides antioxidant properties due to its phenolic compound, and this substance is able to inhibit the inflammation process. The inflammation inhibition resulted in decreased activation of NF-κB and NFATc1. 82 By decreasing the NF-kB, proinflammatory cytokine production, like IL-1β, 54 tumor necrosis factor-alpha (TNF-α), and IL17, 56 decreases. On the other hand, the decrease in NFATc1 expression also affected STAT3 for controlling osteoclast differentiation 83 and bone metabolism 84 to prevent bone resorption 85 and bone loss. 55

**Fig. 4:**
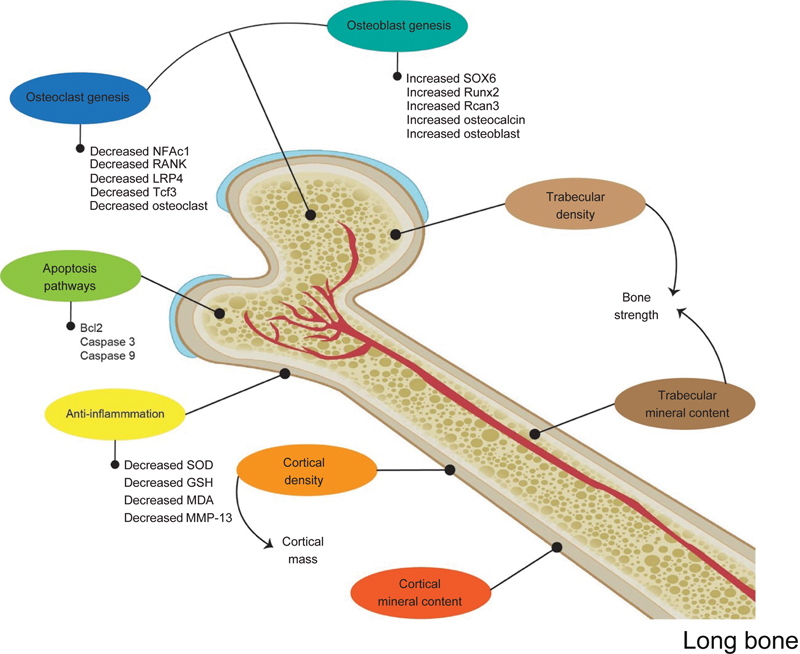
The possible mechanism of grape seed extract supplementation for skeletal bones related to its strength. GSH, glutathione; LRP-4, lipoprotein receptor-related protein 4; MDA, malonaldehyde; MMP-13, matrix metalloproteinase 13.

Low-density lipoprotein receptor-related protein 4 (LRP-4) also decreased after GSE supplementation. Even the exact mechanism of LRP-4 is not fully understood, but also the role of LRP takes place and controls bone morphogenesis. 86 Unlike MMP-13, 54 this protein regulates osteoclast number and activity, bone resorption, and bone mass 80 and maintains mineralization in the bone. 87 By affecting all proteins during bone remodeling, the process of bone regeneration occurs, marked by increased bone volume, 48 trabecular number and thickness, 48 50 and increased bone minerals like phosphor and calcium and also osteocalcin 54 ( Fig. 4 ). By these various effects obtained in the *in vivo* model, it is promising that GSE can be applied to humans in case of bone regeneration, not limited to skeletal bone, but also to jaw bone and alveolar bone.

## Conclusion

Finally, from the available data, we can conclude that the supplementation of GSE affects the alveolar bone, jaw bones, and skeletal bone by promoting bone formation and inhibiting bone resorption by suppressing inflammation, apoptosis pathways, and osteoclastogenesis. It not only supports bone healing in bone inflammation and bone remodeling in osteonecrosis, osteoporosis, and arthritis but also increases bone health by increasing the density and mineral deposition in trabecula and cortical, as well as increases the mineral, calcium, and phosphate deposition. The supplementation of GSE supports bone remodeling by interfering with the inflammation proses and bone formation by preventing bone resorption and maintaining bone health. The evidence in this scoping review gives the opportunity to conduct further research on humans.

## Future Implication

The data presented showed that the GSE has a beneficial effect on human health, particularly in maintaining bone health. Future research should consider the supplementation of GSE not only for bone maintenance but also for treating and supporting bone remodeling in dentistry and orthopaedic treatment. In the field of dentistry, GSE supplementation during the retention phase may prevent postorthodontic relapses by promoting bone regeneration. However, the optimal supplementation dose needs to be determined to achieve a therapeutic effect.

## Limitations

This review was limited by the scarcity of high-quality studies, with most of the available research conducted on animal models or *in vivo* . Additionally, the lack of information regarding the specific doses of GSE used and the duration of the treatment represent significant issues that should be addressed in future studies to enable a more comprehensive meta-analysis.
